# Interaction Between Oxytocin and Dopamine Signaling: Focus on the Striatum

**DOI:** 10.3390/ijms26178711

**Published:** 2025-09-06

**Authors:** Diego Guidolin, Cinzia Tortorella, Chiara Cervetto, Manuela Marcoli, Guido Maura, Luigi F. Agnati

**Affiliations:** 1 Department of Neuroscience, University of Padova, 35122 Padova, Italy; cinzia.tortorella@unipd.it; 2 Department of Pharmacy, University of Genova, 16126 Genova, Italy; cervetto@difar.unige.it (C.C.); manuela.marcoli@unige.it (M.M.); guido.maura@gmail.com (G.M.); 3 Department of Biomedical, Metabolic Sciences and Neuroscience, University of Modena and Reggio Emilia, 41121 Modena, Italy; luigi.agnati@gmail.com

**Keywords:** oxytocin, dopamine, nucleus accumbens, receptor–receptor interaction, receptor complexes, astrocytes

## Abstract

Striatum can be described as a brain region containing a general neuronal mechanism to associate actions or events with reward. In particular, neural activity in the human striatum is modulated by social actions and, critically, by the conjunction of social actions and own reward. To perform this function, dopamine and oxytocin signaling reaching the striatum represent a key factor. These neurotransmitters, in both humans and animals, are released in response to afferent vagal and sensory stimulation, as well as sexual and social interactions, conveying information related to reward and pleasure associated with an event. Dopamine and oxytocin have several effects in common, but of particular interest is evidence indicating that they can mutually modulate their action. The present review focuses on available data delineating interactions between dopaminergic and oxytocinergic signaling in the striatum. In this context, recent data on the possible role played by striatal astrocytes and microglia as key modulators of this crosstalk will be briefly discussed.

## 1. Introduction

The monoamine dopamine is a well-known very important central neurotransmitter with a widespread receptor distribution in the brain and representing the signaling molecule of an extensive neural system (see [1]), whose basic architecture was for the first time described in the 1960s [2,3]. Dopaminergic neuronal cell bodies located in the substantia nigra, hypothalamus, ventral tegmental area, arcuate nucleus, and the zona incerta project to various brain structures forming five main pathways [4,5,6]. They include the nigrostriatal pathway, running from the substantia nigra to the striatum, the mesolimbic/mesocortical pathways, originating in the ventral tegmental area and projecting to ventral striatum and prefrontal cortex, respectively, the tubero-infundibular pathway, from the hypothalamus to the pituitary gland, and the incerto-hypothalamic pathway, reaching the lateral septum and other areas within the hypothalamus. In addition to these pathways within the brain, a pathway from the hypothalamus to the spinal cord is also present (see [7] for a review). Furthermore, there are dopaminergic neurons locally spread, for instance in the paraventricular nucleus of the hypothalamus.

Over the last 30 years, the peptide oxytocin (a classical neurohypophysial hormone) has emerged as an influential signaling molecule released in the brain [8]. It is primarily synthesized in the supraoptic nucleus (SON) and in the paraventricular nucleus (PVN) of the hypothalamus [7]. Axons of the magnocellular neurons in SON and PVN reach the posterior pituitary to release the peptide into the blood, while parvocellular neurons in the PVN project to extrahypothalamic regions of the central nervous system, such as prefrontal cortex, striatum, ventral tegmental area, hippocampus, amygdala, olfactory bulb, brain stem, and spinal cord [9]. Of interest, is also evidence indicating that oxytocin can be released from the dendrites of both the parvo- and the magnocellular neurons in the PVN and SON, allowing for a widespread diffusion of the peptide within the central nervous system [10]. Consistently, oxytocin receptors are also abundantly expressed throughout the CNS. In addition to their expression in the SON and PVN, oxytocin receptors are also found in the abovementioned regions reached by oxytocin fibers [11]. However, some brain areas (e.g., olfactory bulb, ventral pallidum, medial preoptic area and ventromedial nucleus of the hypothalamus) exhibit a distinct mismatch between oxytocin fiber distribution and oxytocin receptor expression [7,12,13], suggesting that centrally released oxytocin can also act as a neuromodulator operating by volume transmission [14,15,16]. Oxytocin receptors are present in the presynaptic and postsynaptic membranes at both excitatory and inhibitory synapses, and the molecular effect of oxytocin on neuronal excitability is the regulation of the activity of ion channels in the cell membrane (see [17] for a review).

The main characteristics of the two signaling systems are summarized in Table 1. As shown, despite their molecular differences, dopamine and oxytocin signaling pathways exhibit a range of functional effects and characteristics in common, being both involved in the regulation of social behaviors, feeding, and reward mechanisms (see [18] for a recent review). Disturbances of these signals are known to be implicated in behavioral disorders including anxiety, depression, autism spectrum disorders (ASD), obsessive–compulsive disorders (OCD), attention deficit hyperactivity disorders (ADHD), and schizophrenia [7,18].

Furthermore, evidence for the existence of dopamine–oxytocin positive interaction in social behavioral paradigms and associated disorders has been reported (see [7] for a review), and anatomical and immunocytochemical studies provided some evidence of possible molecular mechanisms involved in this crosstalk. Receptor binding sites and neuronal fibers of the two neurotransmitters often exist in the same CNS regions of the brain [19,20]. Dopamine receptors, for instance, have been demonstrated in oxytocin neurons, and dopamine signaling influences oxytocin release [21,22]. Conversely, oxytocin has been found to increase dopamine in regions, such as the medial preoptic area, the amygdala, and the ventral tegmental area, where oxytocin receptors are also located [23,24]. Besides modulating each other’s release, the oxytocin receptor and dopamine receptors can also undergo direct receptor–receptor interactions. For instance, both oxytocin and dopamine receptors have been demonstrated in the neurons of nucleus accumbens, and the amygdala [21,23,24], where oxytocin and dopamine D_2_-receptors have been shown to form heteroreceptor complexes leading to changes in the G protein-mediated signal transduction [25,26].

**Table 1:** Main features of dopamine and oxytocin in the brain.

	Dopamine		Oxytocin	
Chemical identity	Monoamine		Nonapeptide	
Main sources	Substantia nigra Ventral tegmental area Zona incerta	[1]	Supraoptic nucleus and Paraventricular nucleus of the hypothalamus	[7]
Main target regions	Dorsal and ventral striatum, Cortex, Hippocampus, Amigdala, Pituitary gland, Hypothalamus, Spinal cord	[4]	Striatum, Prefrontal cortex, Ventral tegmental area, Hippocampus, Amygdala, Brain stem, Spinal cord	[9]
Receptors	D_1_-like (D_1_, D_5_) D_2_-like (D_2_, D_3_, D_4_)	[1,27]	OTR	[22]
Signaling pathway	G_s_ and G_q_ proteins (D_1_-like) G_i/0_ protein (D_2_-like)	[5,28]	G_q_ protein G_i_ protein	[22]
Main effects	Control of organic functions:		Control of organic functions:	
	● Cardiovascular	[29]	● Cardiovascular	[30]
	● Renal	[31]	● Respiration	[7]
	● Penile striated muscles	[32]	● Penile erection	[32]
	Rewarding	[18]	Rewarding	[18]

The present review will focus on available data delineating interactions between dopaminergic and oxytocinergic signaling in the striatum, a region that receives significant dopaminergic and oxytocinergic innervation, and that contributes not only to motor control but also to a broad range of behavioral domains in humans, ranging from basic salience/reward and novelty processing to complex decision making [33,34]. In this context, recent data on the possible role played by striatal astrocytes and microglia as key modulators of this crosstalk will be briefly discussed.

## 2. Dopamine and Oxytocin in the Striatum

The main structure of the basal ganglia was defined as ‘corpus striatum’ in the 17th century because of the mixture of gray matter and fiber tracts it exhibits, and in 1786 Vicq d’Azir was the first who realized it was composed of two main structures, the caudate nucleus and the putamen (see [1]).

From the neuroanatomical standpoint, the striatum is classically divided into (i) dorsal striatum or neostriatum, including most of the caudate and putamen, and (ii) ventral striatum, including the nucleus accumbens, the ventromedial parts of the caudate and the putamen, and the striatal portion of the olfactory tubercle.

### 2.1. Dopamine

Both regions of the striatum receive massive dopamine signals from the midbrain and the understanding of the role of striatal dopamine has developed significantly in recent years (see [35]).

It has been known for a quite long time that the nigrostriatal pathway, running from the substantia nigra (SN) to the dorsal striatum is mainly associated with motor activity, as demonstrated by studies involving lesions [36] or unilateral stimulation [37] of the substantia nigra, and by the motor symptoms associated with Parkinson’s disease. On the other end, ventral striatum receives the mesolimbic pathway, originating in the ventral tegmental area (VTA), projecting to the nucleus accumbens and olfactory tubercle, and involved in reward-related motivation and learning [38]. The claim that dopamine subserves different functions in different striatal regions is also supported by more recent findings [39] showing that dopamine axons in the dorsal striatum preferentially signal locomotion, and those in progressively ventral regions preferentially signal reward. However, a quite complex relationship appears to exist between these two faces of dopaminergic modulation of striatal circuit function. For instance, data exist showing that motor function can still be achieved under conditions of high motivational drive despite dopamine depletion [40], suggesting the dorsal striatum as preferentially stimulating the execution of learned responses rather than motor movements per se. Moreover, recent evidence (see [35,41] for specific reviews) points to dopamine activity in the striatum as mainly conveying information regarding the value of an action, the outcome, and the strength of the outcome–action relationship during goal-directed learning [42,43].

As indicated in Table 1, five different subtypes of dopamine receptors (D_1_, D_2_, D_3_, D_4_, and D_5_) have been identified in brain tissue (see [5] for a review). They belong to the G protein-coupled receptors family, and based on their structure and pharmacological properties, they can be classified into two major groups [27]: D_1_-like receptors (including D_1_ and D_5_) and D_2_-like receptors (comprising D_2_, D_3_, and D_4_). Binding studies have demonstrated some differences between the two groups in terms of affinity to dopamine, with D_2_-like receptors exhibiting a 10- to 100-fold greater affinity to dopamine than D_1_-like receptors [44]. D_1_- and D_2_-like receptors also differ in their genetic structure. D_2_-like receptor genes have introns in their coding regions, while D_1_-like receptor genes do not exhibit this feature [45]. This genetic organization, therefore, enables the generation of D_2_-like receptor splice variants [46]. Concerning signal transduction, it is commonly accepted that the receptors of the D_1_-like group mainly mediate the stimulation of the second messenger adenylyl cyclase (cAMP) by coupling to the G_S_ protein, whereas receptors of the D_2_-like group mainly exert inhibitory effects on this enzyme by coupling to G_i/0_ protein [5,47]. In addition to the main pathway just mentioned, D_1_-like receptors may also couple to the G_q_ protein and modulate phospholipase C [28,48], leading to an increase in intracellular calcium levels and activation of protein kinase C.

All dopamine receptors are expressed in the striatum with abundant levels of D_1_ and D_2_ receptors and moderate expression of D_3_, D_4_, and D_5_ receptors [7]. D_4_ receptors are mainly localized in striatal nerve terminals of glutamatergic neurons localized in the cortex [49,50], and D_5_ receptors are mainly expressed by striatal cholinergic interneurons [51]. In the ventral striatum, striatal efferent medium spiny neurons (MSN) of the nucleus accumbens and the olfactory tubercle express quite high levels of the D_3_ receptor [52]. Very recent data [53] support an important role of this receptor in facilitating memory consolidation following a conditioned stimulus, a function having relevance to conditions (such as generalized anxiety or post-traumatic stress) characterized by a progressive worsening of responses to environmental cues.

D_1_ and D_2_, however, represent the dopamine receptors most abundantly expressed by striatal MSN. D_1_ receptors are mainly expressed by MSN forming the so-called “direct pathway”, while the MSN involved in the so-called “indirect pathway” mainly express D_2_ receptors [54]. Traditionally these two populations are considered to exhibit opposing control over striatal output. This functional segregation, however, is only approximate [55,56]. Despite the distinctive molecular fingerprinting of the two MSN populations, it has been observed that about 10% of the MSN express both D_1_ and D_2_ receptors [54]. Interestingly, it has also been suggested that many of these MSN (more than 90% in the nucleus accumbens and about 25% in dorsal striatum) present D_1_–D_2_ heterodimers, exhibiting pharmacological and signaling properties distinct from the constituent protomers [54,57,58]. Furthermore, D_1_ and D_2_ receptors can also be found pre-synaptically, where they modulate the nerve terminals activity [7]. In this context, of interest is also evidence showing that both D_1_ and D_2_ receptors are often co-expressed with D_4_ or D_5_ receptors [59], and with D_3_ receptors in the ventral striatum [60], where D_1_-D_3_ heterodimers [61] and D_2_–D_3_ heterodimers [62] have also been detected. These findings support the notion that the dichotomous division of MSN according to their expression of D_1_ or D_2_ receptors is not clear-cut [63,64,65].

### 2.2. Oxytocin

Oxytocin parvocellular neurons of the PVN provide the major ascending oxytocinergic pathways reaching forebrain areas, including ventral and dorsal striatum [66,67,68,69]. In these regions, oxytocin terminals appear very sparse when compared to the high density of dopamine terminal networks [15], the highest density of oxytocinergic fibers being observed in the nucleus accumbens [70]. Evidence from animal models (see [71] for a recent review) emphasizes the key role of ventral striatum in behavioral effects of oxytocin, such as social bonding and reward [72,73,74]. Consistently, a reduction in oxytocin receptors in the nucleus accumbens was reported to be associated with aggressive behavior in many species [75,76,77,78]. In humans, the modulatory role of oxytocin on the major striatal pathways has been explored by functional magnetic resonance imaging [79]. This study revealed that oxytocin specifically increased the connectivity between ventral striatum and upstream contralateral cingulate cortex, a circuit involved in reward and motivational processing [33,80,81]. At the same time, however, oxytocin was found to decrease the functional connectivity between putamen and the ipsilateral posterior cerebellar downstream regions, a circuit implicated in motor behavioral control and habit formation [82,83]. These findings, therefore, provide support for the notion that oxytocin in the striatum might shift processing from cerebellar regions involved in habitual responses to frontal pathways involved in context-dependent goal-directed behavior [80].

The oxytocin receptor (OTR) is a 389-amino acid polypeptide belonging to the G protein-coupled receptor family. For signal transduction OTR couples to the G_q_ protein that stimulates the activity of phospholipace C-β isoforms, leading to the generation of inositol trisphosphate (triggering Ca^2+^ release from intracellular stores) and 1,2-diacyl-glicerol (stimulating protein kinase C) [22]. This primary transduction pathway, however, is not the only one triggered by OTR. It is now established that the receptor can also couple to the G_i_ protein [22], leading to different cell responses [84].

OTRs exhibit a variable distribution in the brain of mammals [22] and they have been found in both dorsal and ventral striatum, where a basically complete overlap was observed between OTRs and oxytocin fibers with no significant mismatch [13]. Moreover, in these regions they clearly overlap with the distribution of dopamine D_2_-like receptors [15].

## 3. Oxytocin–Dopamine Interaction in the Striatum

As mentioned before, in both humans and animals, oxytocin and dopamine signals often act together to regulate a variety of social behaviors and habits [15,18,85]. Some of these functional processes involve the striatal circuitry (see [86] for a review).

From the neurophysiological standpoint, striatum is characterized by neuronal activity related to movements, rewards, and the conjunction of both movement and reward [87]. Thus, its neuronal activity is modulated by reward expectation concerning different stimuli, including feeding, sex, pleasure, and social situations [86,88]. In this respect, oxytocin and dopamine play a significant role, being both released in response to these stimuli [89,90,91] and appearing also involved in the mechanisms triggering the escalation of normal behaviors into addiction or abuse, as suggested by studies on side effects of L-dopa [92]. In this context, oxytocin and dopamine are also the most important signals that, acting together, trigger parental behavior. As a matter of fact, after childbirth, an increase in oxytocin receptor density occurs in areas of the brain reached by oxytocin neurons, including ventral striatum (nucleus accumbens in particular) [93,94]. At the same time, dopamine D_2_ receptors increase in the nucleus accumbens [95], and stimulation of D_1_ receptors in this region has been shown to stimulate the onset of maternal behavior in rats [96]. Correlations between maternal behavior, oxytocin, and dopamine levels have also been reported [97]. Of significant interest are also studies [95] in prairie voles (*Microtus ochrogaster*) indicating that the striatum is part of the neural circuitry underlying monogamous pair-bond formation, and that oxytocin and dopamine play a key role in this circuit. Stimulation of D_2_-like receptors in caudate-putamen induces partner preference, while D_1_-like activation prevents pair-bond formation [98,99,100]. Moreover, overexpression of oxytocin receptors in nucleus accumbens was shown to facilitate partner preference [101].

Apart from acting together, oxytocin and dopamine also appear to interact and modulate each other’s effects on striatal activity [7,102]. As indicated by Shahrock et al. [97], for instance, rat dams with a high degree of maternal behavior showed higher levels of dopamine in the nucleus accumbens. Such a condition was abolished following administration of an oxytocin antagonist, suggesting that the oxytocinergic system can regulate the release of dopamine. Consistent with these findings are also human studies based on the application of functional magnetic resonance imaging (fMRI), showing that fMRI signal intensity in the VTA and SN upon viewing a loved partner correlated with the distribution of oxytocin receptor expression [103]. On the other side, studies on drugs of abuse such as cocaine, known to target the mesolimbic dopaminergic system, showed that they significantly reduce the levels of oxytocin in the nucleus accumbens when taken repeatedly over time [104].

Of interest in this context are also available studies on the relationship between striatal oxytocin and dopamine in maladaptive social behavior conditions. Spontaneously hypertensive rats (SHR) have been used as animal models of ADHD [105]. In the nucleus, accumbens of these rats decreased levels of dopamine and changes in dopamine receptors have been reported [106]. Interestingly, SHR also exhibit lower oxytocin levels [107], and pretreatment with oxytocin was able to induce a larger increase in dopamine following methylphenidate treatment, an effect abolished administering an oxytocin antagonist [108]. Animal models of schizophrenia have also shown that oxytocin can modulate the activity in the mesolimbic dopamine pathway. However, in this case oxytocin would decrease the activity, having the capacity to inhibit the hyperactivity in the nucleus accumbens induced by drugs [109]. Several human studies have investigated the interaction between oxytocin and dopamine in the context of ASD, showing that there are links between oxytocin and dopamine also in this condition (see [18,85]). The relationship, however, is not well defined. In a study where improved learning in young adults with ASD was observed as a response to oxytocin, an increase in dopamine signaling in the nucleus accumbens was reported [110]. Studies however are available, reporting both increases and decreases [111,112].

### 3.1. Mechanisms of Interaction

Concerning the mechanisms underlying this mutual modulation of the two signals, different mechanisms can be identified. They will be briefly described here.

#### 3.1.1. Network-Level Mechanism via Interconnected Brain Regions

**Figure 1:**
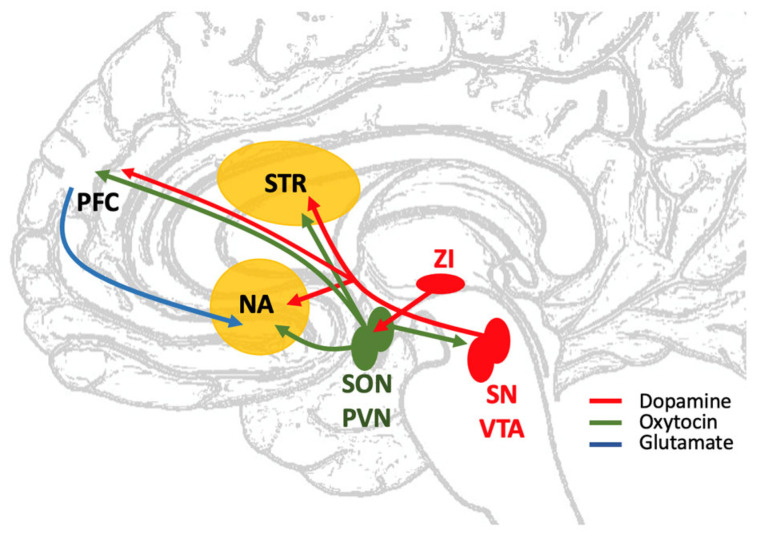
Schematic view of dopaminergic and oxytocinergic pathways involving the striatum [7,15]. STR: dorsal striatum; NA: nucleus accumbens; SN: substantia nigra; VTA: ventral tegmental area; ZI: zona incerta; SON: supraoptic nucleus of the hypothalamus; PVN: paraventricular nucleus of the hypothalamus; PFC: prefrontal cortex. Dopaminergic fibers (red) of the nigro-striatal and mesolimbic pathways coming from SN and VTA target dorsal and ventral striatum. Mesocortical dopaminergic fibers run from VTA to PFC. Oxytocin fibers (green) from parvocellular neurons of PVN also reach the striatum (NA in particular). SON and PVN receive dopaminergic innervation from ZI, and, in turn, send oxytocinergic fibers to SN and VTA. Oxytocinergic fibers also reach neurons of the prefrontal cortex, that send axons (blue) to NA. Their glutamatergic signal could modulate the release of dopamine in this region of the ventral striatum [85].

As schematically illustrated in Figure 1a, first mechanism can be identified in the network of connections between dopaminergic and oxytocinergic regions projecting to the striatum. The dopaminergic incerto-hypothalamic pathway, for instance, projects to the SON and PVN, where oxytocin is produced [113], and dopamine receptors have been demonstrated in these oxytocin neurons [11]. The molecular mechanism behind the modulation of oxytocin release by dopamine is complex and is likely linked to the expression levels of the different dopamine receptor subtypes [18]. Oxytocin neurons mainly express dopamine D_2_ receptors, but D_1_ and D_3_ receptors have also been identified [32], and specific ligands targeting the different subtypes can differently influence the depolarization of the cells [114]. Oxytocin neurons, in turn, project to many areas with dopaminergic neurons, including SN [7,115] and VTA [7,116], where oxytocin receptors have also been identified [22,117].

#### 3.1.2. Indirect Mechanism via Other Neurotransmitters

A second possible mechanism of interaction between oxytocin and dopamine signaling could be mediated by other signaling pathways. Concerning the striatum (see Figure 1), it has been suggested [85] that VTA dopaminergic terminals within the nucleus accumbens express glutamatergic receptors, and that glutamate release from the prefrontal cortex (a region receiving oxytocin fibers [23]) may modulate dopamine release in the accumbens. Supporting the hypothesis, the glutamate-induced release of dopamine has been demonstrated in vitro [118,119], and in vivo [120,121]. Furthermore, oxytocin administration into the prefrontal cortex was shown to induce an increased dopamine release in the nucleus accumbens [122].

#### 3.1.3. Direct Molecular Mechanism via Receptor Complexes

**Figure 2:**
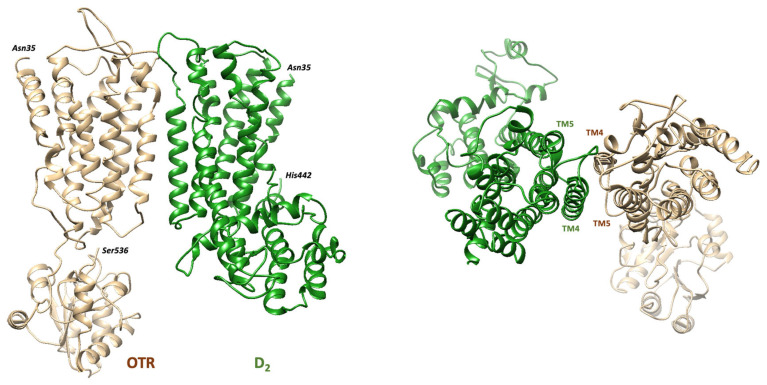
Estimated model of the D_2_-OTR heterodimer. The structure of the complex as predicted by molecular modeling procedures [124] is shown on the (**left**) panel. On the (**right**), a view of the extracellular side is shown to indicate the TM4 and TM5 domains forming the interface between the two protomers.

A third and more direct mechanism of interaction is based on the existence of heteroreceptor complexes between dopamine D_2_ and oxytocin receptors (D_2_-OTR) in the neurons of the nucleus accumbens and the caudate putamen [25,123]. The basic molecular mechanism underlying the formation and the dynamics of these receptor assemblies are allosteric interactions (see [6] for a review). Allostery is a mode of communication between distant sites in a protein, in which the energy associated with dynamic or conformational changes at one site can be transferred (along specific pathways within the protein structure) to other sites, that, in turn, will change their conformational or dynamic features. Thus, when a quaternary structure is established via direct receptor–receptor interactions between protomers, energy perturbations at some site of one protomer can propagate into the nearby protomers and change their conformational and functional properties, leading to a cooperative behavior of the whole complex. Bidirectional facilitatory allosteric receptor–receptor interactions occur in the D_2_-OTR heteromer [26]. In particular, oxytocin-induced OTR activation involves enhancement of D_2_ recognition and signaling [15,26,123]. Figure 2 shows the structure of the heteroreceptor complex as obtained by molecular modeling methods [124]. The residues predicted to be mainly involved in the heteromerization interface are in the transmembrane domains four and five (TM4 and TM5) of both D_2_ and OTR.

Evidence, demonstrating that striatal astrocytes and microglia may represent a key element regulating the interaction between dopamine and oxytocin signaling in the striatum, is a further element of interest. This aspect will be briefly discussed in the section that follows.

## 4. Oxytocin–Dopamine Interactions Mediated by Striatal Astrocytes and a Potential Role for Microglia

In the dorsal striatum, distinct subpopulations of astrocytes, in response to cortical stimulation [125], release glutamate that activates N-methyl-D-aspartate (NMDA) receptors on specific medium spiny neurons and metabotropic glutamate receptors at distal synapses [126]. In the ventral striatum, astrocyte–neuron signaling is also well established as follows: astrocytes respond to neurotransmitters with Ca^2+^ increases and release of gliotransmitters (including glutamate and ATP/adenosine), thereby modulating neuronal activity and synaptic transmission [127]. Reported examples show that D_2_ receptors for dopamine, as well as OTR for oxytocin, may regulate the release of glutamate from the striatal astrocyte processes [128,129].

In this context, of particular interest are quite recent findings demonstrating that D_2_-OTR receptor–receptor interactions (RRI) can be established at the level of the plasma membrane of striatal astrocytes [124]. In this study, confocal imaging showed that both the receptors were expressed on the same astrocyte and astrocyte processes and that activation of either the D_2_ receptor or the OTR could inhibit the evoked glutamate release from the cell, suggesting that both oxytocin and dopamine (through OTR and D_2_ receptors, respectively) possess the capability to regulate glutamatergic transmission in striatal neuron–astrocyte networks. Furthermore, dopamine D_2_ and OTR were found to interact functionally. When oxytocin was bound to its receptor, the affinity of D_2_ receptors increased, allowing to make effective agonist subthreshold concentrations, otherwise too low, to activate the astrocytic D_2_ receptor. As demonstrated by co-immunoprecipitation experiments, this facilitatory RRI was based on receptor heteromerization, a result confirmed by proximity ligation assay [130] tests. Concerning the distribution in the astrocytic cell membrane, lipid rafts appear particularly enriched of these receptor complexes, as suggested by co-immunoprecipitation of oxytocin and dopamine D_2_ receptors with the membrane lipid rafts marker flotillin-1 [131].

The control of glutamatergic transmission in striatum, however, is not the only astrocyte function leading to an interaction between oxytocin and dopamine signaling. Oxytocin was reported to affect the perisynaptic astrocyte processes (PAPs) motility by retracting PAPs and regulating coverage of the synapse [132,133,134], allowing a shift from high privacy of the synaptic transmission (close enwrap of the synapse) to a broad opening of the enwrapping. This would lead to transmitter diffusion (by extra-synaptic volume transmission [135]) to neighboring synapses. Thus, the presence of OTR and D_2_-OTR heteromers on striatal PAPs suggests that oxytocin can be involved in the control of this process. Interestingly, it is known that in striatum, dopamine can also be released non-synaptically and act through volume transmission [136,137,138]. Therefore, the oxytocin’s ability to control astrocytic coverage of the synapses could represent a significant mechanism to modulate dopamine diffusion in striatum, potentially relevant in physiological and pathological conditions.

Available data suggest that also microglial cells are responsive to both oxytocin [139] and dopamine [140], leading to a reduction in the proinflammatory activity of microglia. Dopamine stimulation was found to decrease LPS-induced microglial nitric-oxide production [140], and oxytocin decreases proinflammatory factor levels of TNF-α and IL1-β [141]. In this respect, quite recent functional studies have shown that microglia help organize social circuits and shape social behavior associated with dopamine and oxytocin signaling, as, for instance, pair bonding (see [142] for a specific review). Consistently, neuroinflammation is a component of many disorders associated (see Section 3) with unbalanced dopamine–oxytocin interaction. This process is triggered by microglia in the classical M1 phenotype (see [143]) characterized by the production of proinflammatory factors and the release of glutamate [144]. Altogether these observations may suggest a possible involvement of microglia in the dynamics of the relationship between dopamine and oxytocin. Many aspects of these processes, however, would require additional research to be fully elucidated.

## 5. Concluding Remarks

Striatum can be described as a brain region containing a general neuronal mechanism to associate actions or events with reward. In particular, neural activity in the human striatum is modulated by social actions and, critically, by the conjunction of social actions and own reward [86]. Consistently, striatum is the target of a dense dopaminergic innervation from SN and VTA and of oxytocinergic fibers coming from parvocellular neurons of the hypothalamus. Dopamine and oxytocin are two key neurotransmitters with widespread functions in the brain. They control organic functions, as in the cardiovascular [29,30] system, but in both humans and animals, they are also released in response to afferent vagal and sensory stimulation, as well as sexual and social interactions, representing signals related to reward and pleasure associated with an event [18,102,145,146].

In this respect, dopamine and oxytocin have several effects in common, but of particular interest is evidence indicating that they can mutually modulate their action [7,18]. As discussed in the previous sections, in striatum different mechanisms mediating this interaction between the two signaling systems can be identified. Some of these processes involve dopaminergic or oxytocinergic cell groups projecting to the striatum, whose activity is modulated by signals based on the other neurotransmitter [113]. Other indirect processes involve a different signaling pathway, as in the abovementioned example of the glutamatergic cortico-striatal pathway, modulated by oxytocin signals and regulating dopamine release in the striatum [85]. Direct processes also exist, involving striatal cells and synapses. Examples include the formation of D_2_-OTR heteroreceptor complexes in striatal neurons and astrocytes [123,124], and the regulation by striatal astrocytes of dopaminergic volume transmission [124,132].

These mechanisms attracted attention also from a therapeutic standpoint. Of particular interest in this field are studies exploring strategies targeting oxytocinergic signaling to regulate dopaminergic transmission when altered. The dynamics of the D_2_-OTR heteroreceptor complex provides an example (see [134]). In this, heteromer oxytocin has a facilitatory effect on D_2_ receptor activation. Then, it might help to maintain dopaminergic neuron function in early Parkinson’s disease (PD), to delay the onset of PD symptoms related to defective dopamine receptor activation, and to make effective low doses of PD medications. Thus, the potential clinical usefulness of oxytocin as adjunctive drug therapy in PD patients would be based also on the possibility to reduce the dopaminergic therapy side effects. As a matter of fact, some evidence exists that after intranasal administration, oxytocin concentration increased in the striatum [147], and dopamine levels also increased [148], improving locomotor disabilities and anxiety-like behavior [149]. However, although in many studies oxytocin was shown to increase dopaminergic activity, evidence also exist identifying conditions in which oxytocin treatment has the capacity to decrease dopaminergic activity, as, for instance, in animal models of schizophrenia, where oxytocin seems to decrease dopaminergic activity in the nucleus accumbens [109].

Various mechanisms have been proposed to explain the observed opposite effects of oxytocin on dopaminergic activity. The variability in the oxytocin gene, as suggested by Love et al. [150], is a first possibility. In addition, very recently it has been reported that in striatal astrocytes oxytocin could induce dual responses, namely the inhibition and facilitation of both Ca^2+^ signals and glutamate release, and that the inhibitory and the facilitatory response appear dependent on activation by OTR of different transduction pathways, the G_i_ and the G_q_ pathway, respectively [151]. It is also possible that the many and sometime opposite effects might be explained by the expression levels of the different subtypes of dopamine receptors, and the possible different receptor complexes they could form [25,26]. The different effects, of course, may also be a consequence of the complex network of integrative processes [152] involving oxytocin and dopamine systems in the brain, and of the interactions they may have with other signaling lines [18,26]. Thus, this complex pattern of different and sometimes opposite effects makes it presently difficult to use dopamine and oxytocin (or analogs) as treatments.

The challenge is to find more specific agents acting on specific elements and processes mediating dopamine–oxytocin interactions. In this respect, receptor complexes involving dopamine receptors and OTR may represent a promising research line [26]. In view of the demonstrated existence of many heterodimers involving dopamine D_3_ receptor [61,153,154,155], of particular interest may be to test if also D_3_-OTR complexes exist in ventral striatum, and their involvement in striatal functional processes. The OTR transduction pathways certainly represent a second research objective. In view of the diverse effects the oxytocin receptor can induce [151] depending on the G protein it exploits, exploring possible strategies to modulate the transduction pathway may be a target of pharmacological interest. Finally, to expand our understanding of dopamine–oxytocin interaction, it is important to consider not only neuronal dynamics, but also the role and the contribution of glial cells. As briefly outlined here, recent data support a significant role of striatal astrocytes in the regulation of the interaction between dopamine and oxytocin signaling, and functional studies have been reported [142] suggesting that also microglial cells may have the capacity to be regulators of these processes. Specific studies on microglia, however, are still at the beginning and more research activity would be needed to characterize the potential regulatory mechanisms associated with these cells. A research effort applied to glial cells, however, may represent a topic of particular interest, not only to reach a better understanding of the role they have in the regulation of dopaminergic and oxytocinergic systems, but also from a therapeutic standpoint. Such a research effort, indeed, may open the possibility of exploring novel, glia-mediated, strategies to address the disorders associated with unbalance of these signals [156].

## Data Availability

Data sharing is not applicable since no new data were recorded or analyzed in this study.

## Abbreviations

The following abbreviations are used in this manuscript:

SON	Supraoptic nucleus
PVN	Paraventricular nucleus
SN	Substantia nigra
VTA	Ventral tegmental area
ASD	Autism spectrum disorder
ADHD	Attention deficit hyperactivity disorder
MSN	Medium spiny neurons
OTR	Oxytocin receptor
SHR	Spontaneously hypertensive rats
TM	Transmembrane domain
RRI	Receptor-receptor interaction
PAP	Perisynaptic astrocyte process
